# Deep Eutectic Solvents and Wall-Breaking Technique: A New Frontier in the Extraction of Oleuropein and Flavonoids from Olive Leaves with Superior Antioxidant and Antitumor Potential

**DOI:** 10.3390/molecules30051150

**Published:** 2025-03-03

**Authors:** Yan Deng, Junlin Zhou, Jipen Qu, Bixia Wang, Xiao Xu, Chunyan Zhao

**Affiliations:** 1 College of Life Science, Environmental Science and Engineering, China West Normal University, Nanchong 637009, China; 18121927870@163.com (Y.D.); 18282097686@163.com (J.Z.); 2 College of Agricultural Science, Xichang University, Xichang 615000, China; ququ8312@163.com; 3 Sichuan Yizhang Agricultural Development Co., Ltd., Nanchong 637009, China; 17883305560@163.com

**Keywords:** olive leaves, oleuropein, luteolin-7-O-glucoside, wall-breaking extraction, antioxidant activity, antitumor activity

## Abstract

The main objectives of this study were to develop an optimized green extraction process to obtain high contents of oleuropein and flavonoids from olive leaves. A deep eutectic solvent (DES) combined with wall-breaking extraction (WBE) was employed. A DES composed of choline chloride and ethylene glycol in a 1:2 molar ratio with 30% moisture content outperformed lactic acid and methanol as extraction solvents. The optimal conditions, determined by response surface methodology, were 30% moisture content, 140 s of wall-breaking time, and a 230 mL/g liquid–solid ratio. Under these conditions, 88.87 mg/g DM oleuropein, 4.57 mg/g DM luteolin-7-O-glucoside, and 114.31 mg RE/g total flavonoids were obtained. Among three olive varieties (Arbosana, Arbequina, and Picholine) cultivated in China, young Picholine leaves exhibited the highest contents. The Picholine-enriched extract demonstrated higher antioxidant activity (ABTS^•+^ 155.10 mg/mL, DPPH^•^ 44.58 mg/mL) compared to other DES-based extracts, although it was lower than that of purified compounds. Furthermore, the CCK-8 assay revealed significant inhibition of Eca-109 human esophageal cells by the Picholine-enriched extract at 25 µg/mL for 24 h, compared to Het-1A cells. This process effectively recovers bioactive compounds from olive by-product, and shows potential for applications in nutritional supplements, cosmetics, and the food industry.

## 1. Introduction

Olive (*Olea europaea* L.) is a cash crop, and with the growing demand for olive oil and its health benefits, the olive industry is expanding, leading to increased by-product production. Olive leaves, a significant agricultural by-product, yield about 25 kg per tree including twigs [1], representing 10% of olives processed and contributing to 4.5 million tons of global waste annually [2], with China generating over 600,000 tons [3,4]. The lack of effective recycling methods has led to environmentally harmful practices, highlighting the need for sustainable management.

Related studies have shown that olive leaves are rich in bioactive compounds, notably secoiridoids and flavonoids [4,5]. Oleuropein (OE) is the predominant secoiridoid, while luteolin-7-O-glucoside (LE) is the main flavonoid, both known for their multitude of health benefits [6], including antioxidant, antimicrobial, anti-cancer, anti-inflammatory, and neuroprotective properties [7,8,9]. The remarkable biological activities of OE and LE have spurred extensive research into the content variations of these compounds across different varieties and origins [10,11]. Concentrations of OE and LE can reach up to 441 g/kg and 11 g/kg in dry leaves, respectively [5,12]. Various extraction techniques have been explored, with Lama-Muñoz et al. [6] having utilized ultrasound-assisted extraction (UAE) and attained optimal yields of 69.91 g/kg for OE and 1.82 g/kg for LE. These compounds hold potential functional, pharmacological, nutraceutical, and cosmeceutical values [12,13].

Selecting the appropriate extraction technique is essential to fully exploit the potential of these bioactive compounds. Conventional methods such as microwave-assisted extraction [14], UAE [6], supercritical fluid extraction [15], and pressurized liquid extraction (PLE) [16] have been employed. However, these techniques often require high temperatures and pressures, potentially causing compound degradation and reduced preservation effects. For instance, Lama-Muñoz et al. [6] applied PLE to simultaneously extract oleuropein and luteolin-7-O-glucoside from olive leaves at temperatures up to 190 °C. Given the sensitivity of oleuropein and flavonoid aglycones to high temperatures, this poses a significant challenge. To address this, mechanical wall-breaking extraction (WBE) has emerged as a sustainable alternative for extracting low-concentration compounds at room temperature (25 °C) [17]. This physical method uses multiple sets of high-speed rotating blades to cut the sample, disrupting the plant cell wall and rapidly releasing active substances [18]. WBE is particularly effective for isolating naturally occurring low-concentration compounds, such as flavonoids, oleuropein, and saponins, which are crucial for preserving the therapeutic properties of natural products in pharmaceutical and nutraceutical applications [3].

In extraction processes, solvents like water, ethanol, acetone, and ethyl acetate are commonly used to isolate polyphenols from olive leaves [19]. Nevertheless, these solvents pose significant toxicity and environmental concerns. As a more eco-friendly option, deep eutectic solvents (DES) have gained attention [20]. DESs are created through the combination of hydrogen bond acceptors (HBAs) and hydrogen bond donors (HBDs), facilitating the selective extraction of various compounds from a single biomass source [21]. Choline chloride ([Ch]Cl), a commonly utilized HBA, is often paired with HBDs like lactic acid, sugars, organic acids, or urea to solubilize both non-polar and polar compounds [22,23]. Research by García et al. [23] demonstrated that effectiveness of DES, combined with [Ch]Cl and various compounds like sugars, alcohols, and urea, could effectively extract phenolic compounds from virgin olive oil. Similarly, De Almeida Pontes et al. [19] successfully extracted phenolic compounds from olive leaves using a DES composed of [Ch]Cl and carboxylic acids, outperforming traditional ethanol-based methods. A notable limitation of these DES-based solvents is their high viscosity, often requiring the addition of water to adjust the consistency [24]. To date, there has been limited research on DES-based WBE of bioactive compounds from olive leaves.

In this study, DES combined with WBE was employed to optimize the yields of oleuropein (OEY), luteolin-7-O-glucoside (LEY), and total flavonoid content (TFC) from olive leaves. Firstly, a comprehensive screening was conducted to determine the optimal composition of DES. Subsequently, response surface methodology (RSM) was used to optimize three key parameters in the DES-WBE process: wall-breaking time, DES moisture content, and liquid–solid ratio. Next, we analyzed the content of active ingredients in both old and young leaves of the major olive varieties cultivated in China. Additionally, the antioxidant potential of the extracts enriched in oleuropein and luteolin-7-O-glucoside (EOL) was assessed using two standard free radical scavenging assays: 2,2-diphenyl-1-picrylhydrazyl (DPPH^•^) and 2,2′-azino-bis(3-ethylbenzothiazoline-6-sulfonic acid) (ABTS^•+^). Furthermore, the cell counting kit-8 (CCK-8) method was utilized to evaluate the effects of the enriched extracts on the viability of esophageal cancer cells (Eca-109) and normal esophageal cells (Het-1A), providing insights into their cytotoxicity and impact on cell viability.

## 2. Results and Discussion

### 2.1. Screening of DESs for High Extraction Efficiency

To identify efficient solvents for extracting oleuropein and flavonoids, various DES combinations and moisture content were screened at room temperature (25 °C) using a WBE power of 800 W, a wall-breaking time of 120 s, and a liquid–solid ratio of 200 mL/g. Based on the literature and preliminary experiments [21,25], eight DES types with varying ratios were evaluated (Table 1). DES-5 to DES-8, utilizing ethylene glycol as the HBD, exhibited OEY, LEY, and TFC values of 53.19–58.58 mg/g DM. 3.73–4.11 mg/g DM, and 80.88–98.82 mg RE/g, respectively (Table 2). In contrast, DES-1 to DES-4 with lactic acid as HBD displayed different values, notably very low TFC. DES-6, formulated with a 1:2 molar ratio of [Ch]Cl/ethylene glycol, stood out with an OEY of 58.58 mg/g DM, an LEY of 4.11 mg/g DM, and a TFC of 98.82 mg RE/g (Table 2), highlighting the superiority of glycol-based DES over lactic acid-based DES, consistent with findings on elderberry and rosemary [26]. Thus, DES-6 was deemed the optimal formulation.

**Figure 1:**
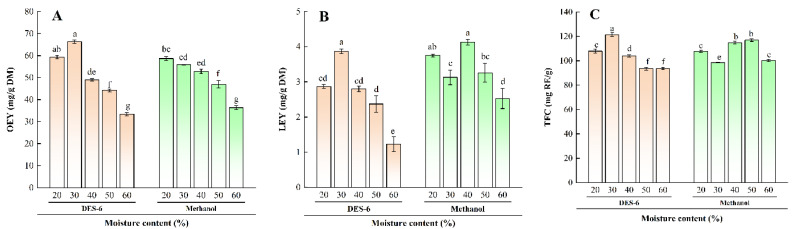
Comparison of OEY, LEY, and TFC extraction using DESs with varying moisture contents versus methanol in WBE. (**A**) OEY content; (**B**) LEY content; (**C**) TFC content. OEY, oleuropein yield; LEY, luteolin-7-O-glucoside yield; TFC, total flavonoid content. Statistically significant differences in OEY and flavonoids extracted using various DES solvents are indicated by different lowercase letters.

The viscosity of DES can impact extraction efficiency [26]. High viscosity impedes the application of DES in extraction processes [27]. Generally, DES viscosity increases with a higher molar ratio of HBA to HBD [28]. Five moisture contents (20–60%) were investigated, with methanol serving as a comparison. As moisture content increased, OEY, LEY, and TFC varied from 33.44 to 66.29 mg/g DM, 1.23 to 3.87 mg/g DM, and 93.53 to 121.42 mg RE/g, respectively (Figure 1A–C). The moisture content of methanol also significantly affected the yields, especially LEY. DES outperformed methanol, with maximum OEY, LEY, and TFC at 30% moisture content: 66.29 mg/g DM, 3.87 mg/g DM, and 121.42 mg RE/g, respectively. Consistent with the literature [21], a water content of 20–40% was optimal. Therefore, DES with 30% moisture content was selected for subsequent experiments.

### 2.2. Exploring of Key Influencing Factors Using Single-Factor Experiments

**Figure 2:**
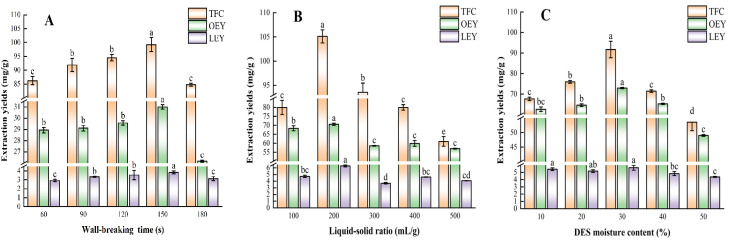
Effect of different (**A**) wall-breaking time, (**B**) liquid–solid ration, (**C**) DES moisture content on OEY, LEY, and TFC. In the same single-factor experiment, statistically significant differences exist between the same component under different conditions, which are indicated by different lowercase letters.

After obtaining the optimal DES, we explored the effects of wall-breaking time (A), liquid–solid ratio (B), and DES moisture content (C) on extraction efficiency through single-factor experiments. Figure 2A–C displays their impact on OEY, LEY, and TFC during the WBE process. The wall-breaking time of plant cell wall significantly affects flavonoids [29]. Figure 2A investigates how wall-breaking times (60–180 s) affect OEY, LEY, and TFC, with DES moisture at 30% and a ratio of 200 mL/g. OEY peaked at 31.00 mg/g DM at 150 s, then declined. LEY and TFC also peaked at 150 s, at 3.83 mg/g DM and 99.24 mg RE/g, respectively. This suggests that disrupting the cell wall at 150 s enhances solubility in DES [30], providing insights for subsequent optimization.

The liquid–solid ratio during wall-breaking extraction is one of the key factors affecting the extraction efficiency [29]. Figure 2B analyzes the effect of liquid–solid ratios (100–500 mL/g) on OEY, LEY, and TFC, with a fixed wall-breaking time of 150 s and 20% DES moisture. TFC and OEY reached their maximum yields at 200 mL/g, 105.11 mg RE/g and 70.57 mg/g DM, respectively, then declined. LEY followed a similar trend. This optimal ratio may be due to changes in density and viscosity, enhancing the release of oleuropein and flavonoids [19].

Due to DES’s high viscosity, ultrapure water was added to reduce it [24]. Figure 2C illustrates the impact of DES moisture content (10–50%) on OEY, LEY, and TFC, with a constant time of 150 s and ratio of 200 mL/g. LEY peaked at moisture contents of 10% (5.63 mg/g DM) and 30% (5.43 mg/g DM). Meanwhile, OEY and TFC reached their maximum values at 30% moisture content, with values of 72.93 mg/g DM and 91.71 mg RE/g, respectively. The incorporation of 30% water likely reduced DES viscosity, enhancing extraction efficiency [26]. These results indicate that a 30% DES moisture content is optimal for maximizing OEY, LEY, and TFC, serving as a crucial reference for subsequent experiments.

### 2.3. Optimization of DES-Based WBE Conditions Through Response Surface Methodology

#### 2.3.1. Box-Behnken Design and Model Fitting

Drawing from the outcomes of the single-factor experiments, the independent variables selected were A (wall-breaking time), B (liquid-solid ratio), and C (moisture content), which were assigned codes of −1, 0, and 1, respectively. A combined design experiment was conducted using Design Expert 10 software following the BBD model. After 17 rounds of tests, the effects of the three-factor interactions on OEY, LEY, and TFC were determined, and are shown in Table 3. The response values of OEY, LEY, and TFC ranged from 69.05 to 86.71 mg/g DM, 2.73 to 4.35 mg/g DM, and 67.97 to 114.90 mg RE/g, respectively. The independent variables (Y_OEY_, Y_LEY_, and Y_TFC_) were found to have significant effects, as indicated by the results. A second-order polynomial equation, derived from multiple regression analysis of the experimental data and expressed in terms of coded variables, is presented as follows:

Y_TFC_ = 108.61 − 3.96A − 5.18B − 4.88C − 6.70AB + 0.94AC − 2.11BC − 7.42A^2^ − 12.95B^2^ − 12.62C^2^

Y_OEY_ = 8.64 − 0.14A + 0.11B − 0.034C − 0.057AB + 0.31AC − 0.23BC − 0.41A^2^ − 0.42B^2^ − 0.93C^2^

Y_LEY_ = 0.42 − 0.023A + 0.011B + 0.012C − 0.015AB + 0.024AC − 0.0086BC − 0.031A^2^ − 0.017B^2^ − 0.061C^2^

#### 2.3.2. Confirmation of Adequacy of Fit and Influence Analysis

The analysis of variance (ANOVA) results demonstrated the reliability of the second-order polynomial models [31]. Table 4 presents a summary of the ANOVA results for the polynomial model of response surface, which includes all the independent variables. The F-values for the OEY and LEY models are 463.65 and 71.38, respectively, with corresponding *p*-values < 0.0001, indicating highly significant models [29]. The TFC model has an F-value of 9.03 and a *p*-value of 0.0042, also demonstrating statistical significance. The lack of fit for OEY, LEY, and TFC was deemed insignificant, as evidenced by *p*-values of 0.0871, 0.6252, and 0.4736 (*p* > 0.05), confirming a very satisfactory fit of the model to the experimental data [16]. Furthermore, the adjusted coefficients of determination (Adj-R^2^) for the OEY, LEY, and TFC models were determined to be 0.9962, 0.9754, and 0.8188, respectively, with corresponding coefficients of variation (CV) of 0.50%, 1.92%, and 1.90%. These figures suggest a high level of reliability and accuracy in the results.

For the OEY model, the linear coefficients (A and B), interaction terms (AC and BC), and quadratic terms (A^2^, B^2^, and C^2^) exerted the most significant negative influence (*p* < 0.0001). Additionally, the factors C and AB were identified as crucial in determining OEY. In the case of the LEY response, the linear coefficient A and quadratic terms (A^2^ and C^2^) were found to have a substantial impact, while B, C, AB, AC, BC, and B^2^ also contributed notably. For TFC, the linear coefficients (B and C), the interaction term (AB), and the quadratic terms (A^2^, B^2^, and C^2^) were statistically significant. However, the interaction terms (AC and BC) did not exhibit any discernible effect on TFC. Based on the *p*-values, it is evident that both the ratio (B) and the moisture content (C) had considerable influences on all three parameters.

#### 2.3.3. Effects of Extraction Parameters on Responses

**Figure 3:**
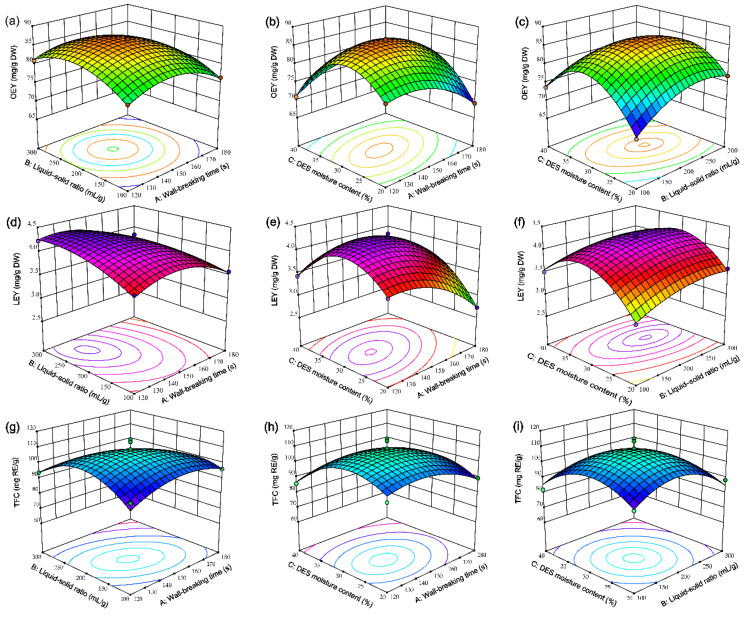
3D response surface plots illustrating the impact of independent variables on OEY (**a**–**c**), LEY (**d**–**f**), and TFC (**g**–**i**): (**a**,**d**,**g**) Interaction between wall-breaking time (A) and liquid-solid ratio (B); (**b**,**e**,**h**) Interaction between wall-breaking time (A) and DES moisture content (C); (**c**,**f**,**i**) Interaction between liquid-solid ratio (B) and DES moisture content (C).

The 3D (three-dimensional) response surface curve effectively visualized the effects of independent variables and their interactions [32]. Specifically, Figure 3a–i depicted how three independent factors—wall-breaking time (A), liquid–solid ratio (B), and moisture content (C)—influenced OEY, LEY, and TFC. When the time (A) was within the range of 100 to 150 s and the ratio (B) varied from 100 to 200 mL/g, the interaction between A and B exerted a positive influence on OEY, reaching a maximum value of 86.71 mg/g DW at 150 mL/g and 200 s (Figure 3a). The contour map for the A and C interaction was elliptical, indicating that excessively long times and high moisture content adversely affected OEY (Figure 3b). Similarly, when the time was 150 s, the steep surface of the BC interaction contour map demonstrated a significant effect on OEY (Figure 3c).

Figure 3d illustrated that the interaction between A and B had a notable impact on LEY. With a constant moisture content of DES, an extension in wall-breaking time (A) and an increase in the ratio (B) were observed. Within the ranges of 120–150 s and 100–200 mL/g, LEY rose from 0.43 mg/g DM to 2.73 mg/g DM. However, LEY experienced a considerable decline in the intervals of 150–180 s and 200–300 mL/g. The trend in the interaction of A and B mirrored this pattern. Specifically, when the time was adjusted to 150 s and the moisture content reached 30%, LEY attained a peak value of 4.35 mg/g DM (Figure 3e). With the time held constant at 150 s, LEY exhibited an upward trend as the liquid–solid ratio and moisture content increased, yet it began to decline gradually beyond the thresholds of 200 mL/g and 30%, respectively (Figure 3f).

However, Figure 3g–i demonstrated that TFC was differentially influenced by the interplay of the three independent variables. The contour lines depicting the interaction of A and B were elliptical, suggesting that the interaction between A and B had an impact on TFC [33]. Furthermore, TFC rose as the time extended and the ratio increased, reaching a peak of 114.90 mg RE/g at 150 s and 200 mL/g (Figure 3g). Conversely, the contour lines representing the interactions between moisture content and both the time and liquid–solid ratio (AC and BC) were circular, with a relatively flat interaction surface, indicating that these interactions had a minimal effect on TFC (Figure 3h,i).

#### 2.3.4. Verification of the Optimal Predicted DES-Based WBE Conditions

The reliability of the RSM design was further confirmed through 3D surface plotting and regression analysis of independent variables [6]. The verification experiment revealed optimal conditions for OEY and LEY extraction: a time of 141.46 s, a ratio of 220.57 mL/g, and a moisture content of 29.68% (Table 5). For TFC, the optimal conditions were 143.35 s, 187.21 mL/g, and 28.09%. To streamline operations and achieve a balance in the extraction of oleuropein and flavonoids, the actual conditions were set at a wall-breaking time of 140 s, a ratio of 230 mL/g, and a water content of 30% (Table 5). The actual yields obtained were 88.87 mg/g DM for OEY, 4.57 mg/g DM for LEY, and 114.31 mg RE/g for TFC, respectively.

### 2.4. Quantitative Analysis of Oleuropein and Flavonoids in Chinese Olive Leaves

**Figure 4:**
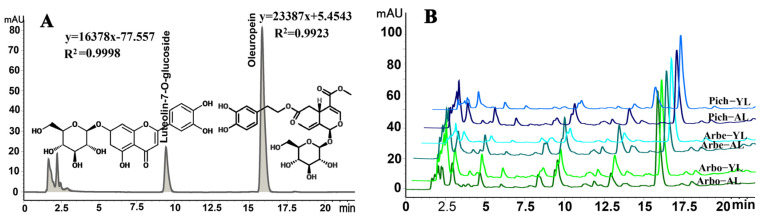
HPLC profiles of oleuropein and luteolin-7-O-glucoside in olive leaves standards (**A**) and samples (**B**). AL, aged leaves; YL, young leaves; Arbo, Arbosana; Arbe, Arbequina; Pich, Picholine.

Based on the analysis method for olive leaves flavonoids described by Meirinhos et al. [34] and combined with the HPLC conditions explored previously in our laboratory’s research [3], the HPLC chromatograms of oleuropein and luteolin-7-O-glucoside, obtained through an optimization experiment, are presented in Figure 4A. Figure 4B displays these compounds in three main Chinese olive varieties. The quantitative results are summarized in Table 6.

Table 6 presented the variations in the OEY, LEY, and TFC across old and young leaves of three olive varieties, ranging from 52.96 mg/g DM to 11.79 mg/g DM for OEY, 4.62 mg/g DM to 1.72 mg/g DM for LEY, and 76.98 mg RE/g to 27.93 mg RE/g for TFC, respectively. These findings suggested that both variety and leaf age significantly affected the levels of oleuropein and flavonoids, consistent with our previous observation that Liangshan olive leaves exhibited variations in phenolic profiles based on these factors [3]. Specifically, young leaves of Picholine demonstrated the highest OEY and TFC, reaching 52.96 mg/g DM and 76.98 mg RE/g, respectively. Conversely, young leaves of Arbosana accumulated the highest LEY at 4.62 mg/g DM, with its old leaves also showing a considerable LEY content of 3.58 mg/g DM. Notably, young leaves of Arbequina maintained high levels of all three compounds, with OEY, LEY, and TFC at 46.59 mg/g DM, 3.57 mg/g DM, and 63.47 mg RE/g, respectively (Table 6). Our findings align with those of Ranalli et al. [35], who emphasized the impact of color/age and genetic factors on oleuropein content. Specifically, our results showed that young leaves typically exhibit higher levels of OE and flavonoids than old leaves. Furthermore, Hashemi et al. [36] reported cultivar- and season-specific variations in oleuropein concentration in their study involving different cultivars and seasons. Consequently, among the varieties examined, young leaves of Picholine emerged as the most promising source of natural flavonoids.

### 2.5. Evaluation of Antioxidant Activity

To assess the antioxidant activity of EOL-enriched extracts from different olive varieties (Picholine, Arbequina, and Arbosana), young leaves were selected for evaluation. OE (70%) and LE (50%) served as benchmarks. Two widely recognized in vitro methods, ABTS^•+^ and DPPH^•^ radical scavenging assays, were employed [37,38]. Table 7 shows that the ABTS^•+^ radical scavenging effect of EOL-rich extracts from Picholine, Arbequina, and Arbosana varieties increased from 14.66% to 51.78%, 16.37% to 56.76%, and 16.51% to 58.14%, respectively, as the concentration increased from 20 to 120 mg/mL. However, these extracts exhibited significantly lower activities than the control vitamin C (Vc) (≈99%). The EC50 value, indicating the concentration needed for 50% antioxidant effect, is a standard measure for comparing antioxidant capacities of different compounds [39]. The EC50 value of Vc was determined to be 3.59 mg/mL. Notably, The EC50 values of OE and LE were significantly lower, at 1.15 mg/mL and 2.41 mg/mL, respectively, indicating their potent antioxidant activity [38], which is comparable to that of Vc [40]. Among the three olive varieties, the EC50 values were Picholine (155.10 mg/mL) < Arbeqina (163.68 mg/mL) < Arbosana (180.97 mg/mL). Overall, although the scavenging capacity of the three samples was inferior to that of Vc, OE, and LE, the Picholine variety exhibited higher activity than Arbequina and Arbosana. This aligns with findings by Topuz et al. [41], highlighting the influence of total phenol content and OEY in olive leaves on ABTS^•+^ radical scavenging.

The DPPH^•^ assay is another reliable method for assessing antioxidant capacity [39]. As shown in Table 7, within a concentration range of 20–120 mg/mL, the DPPH^•^ radical scavenging activity of EOL-rich extracts increased from 26.38% to 68.14% for Picholine, 36.88% to 86.47% for Arbequina, and 31.31% to 82.47% for Arbosana, in a concentration-dependent manner. Lower EC50 values indicate stronger scavenging abilities [38]. Vc and OE exhibited low values of 2.76 and 5.97 mg/mL, respectively, while LE was even lower (1.37 mg/mL). The values for the three samples were as follows: Arbequina (33.43 mg/mL) < Picholine (44.58 mg/mL) < Arbosana (68.82 mg/mL), all lower than Vc. Collectively, the DPPH^•^ scavenging abilities of Vc and OE were similar, with LE being slightly lower, albeit the differences in concentration were minimal. The DPPH^•^ scavenging abilities of the three samples demonstrated significant concentration dependence, with both Arbequina and Picholine extracts showing strong scavenging activity, suggesting that flavonoid levels, particularly TFC, influenced their antioxidant activity [42].

### 2.6. Evaluation of Antitumor Activity

**Figure 5:**
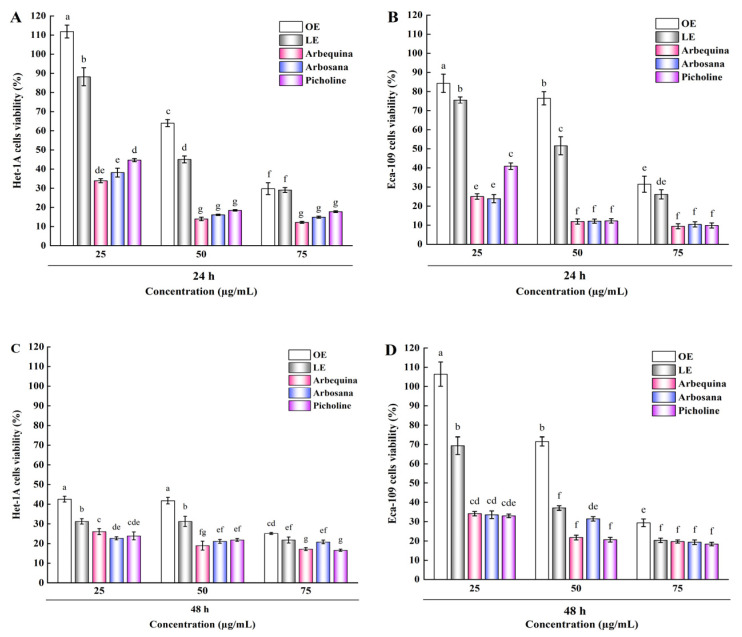
The effect of EOL-rich extracts (25, 50, and 75 µg/mL) on the cell viability of human esophageal cells. (**A**,**B**) cell viability of Het-1A and Eca-109 cells after 24 h of exposure to varying doses; (**C**,**D**) cell viability of Het-1A and Eca-109 cells after 48 h of exposure to varying doses. Different lowercase letters indicate that different components within the same concentration range have varying effects on cell viability.

The antitumor effects of EOL-rich extracts from young leaves of three olive varieties (Picholine, Arbequina, and Arbosana) were investigated in human esophageal cell lines, Eca-109 and Het-1A. Cells were exposed to extracts at 25, 50, and 75 µg/mL for 24 and 48 h, and cell viability was determined using the CCK-8 assay. Results were compared with OE (70%) and LE (50%). Figure 5A,B illustrates that, after 24 h of incubation, the EOL-enriched extract induced a dose-dependent decrease in the viability of Eca-109 and Het-1A cells. Specifically, at 25 µg/mL, OE and LE significantly increased the viability of Het-1A cells (111.85% and 88.21%, respectively) while decreasing the viability of Eca-109 cells (84.30% and 75.47%, respectively). The Arbequina and Arbosana samples exhibited similar selectivity, with Het-1A cell viability exceeding 30% and Eca-109 cell viability remaining below 25%. When treated with Picholine extract from the same donor, both cell lines maintained 40.90% viability. Although cell viability remained above 45% when treated with 50 µg/mL OE and LE, it dropped below 20% when treated with all three samples, indicating a loss of selective cytotoxicity. Furthermore, at 75 µg/mL, the viability of both cell lines decreased significantly without species selectivity. This decline is likely due to the strong cytotoxicity of oleuropein, which has also been observed in other cell lines, such as MDA-MB-231 and MCF-7 cells [43]. These findings corroborate previous results where olive leaf extracts promoted Het-1A growth and inhibited Eca-109 viability at 25 µg/mL for 24 h [29].

Figure 5C,D illustrate the 48 h results. With the extended treatment duration, even at a low dose (25 µg/mL), OE and LE strongly stimulated Het-1A cells, reducing their viability to 42.55% and 31.24%, respectively, while Eca-109 cells maintained higher viability at 106.38% and 69.33%. Similar trends were observed in both cell lines when treated with the three samples. When the doses of OE and LE were increased to 50 µg/mL, Eca-109 cells exhibited greater sensitivity than Het-1A cells, particularly after LE treatment, with cell viability sharply decreasing to 37.06%. This indicates that luteolin-7-O-glucoside possesses potent anti-tumor activity. However, under the same concentration conditions, no significant selectivity was observed between the two cells lines treated with the three samples. This non-selective cytotoxicity is similar to that reported for *Cuminum cyminum* fruits compounds against the MCF-7 cell line, likely due to the high selectivity index (SI) of luteolin-7-O-glucoside (SI = 8.0) [44]. Goodarzi et al. [44] also found that luteolin-7-O-glucoside and apigenin-7-O-glucoside exhibited non-selective effects on the MDA-MB-231 cell line. Additionally, high doses (75 µg/mL) strongly inhibited the survival of both Eca-109 and Het-1A cells, with survival rates falling below 30%. The mechanism underlying the non-selectivity of these two cell lines for the tested samples requires further in-depth study. A similar study reported contrasting effects of olive leaf extracts from Spain and Greece on A375 melanoma cells and HaCaT keratinocytes after 48 h [8]. In summary, EOL-rich extracts significantly affected cell viability, with the Picholine sample demonstrating strong antitumor potential by inhibiting Eca-109 cells and promoting Het-1A cell growth at 25 µg/mL for 24 h.

## 3. Materials and Methods

### 3.1. Materials and Chemicals

The three primary olive varieties, namely Arbequina, Picholine, and Arbosana, were cultivated at the Yizhang Agricultural Olive Garden in Yingshan County, Sichuan Province, China, and harvested in January 2024. The leaves were subsequently dried at 45 °C and then finely powdered using a Daluhong high-speed traditional Chinese medicine grinder, (DC-100-DC-2000, Ningbo, China). For the optimization experiment, young leaves of the Picholine variety were selected. The powdered samples were stored in sealed containers in a desiccator at room temperature (25 °C) until extraction.

Analytical-grade choline chloride, lactic acid, and ethylene glycol were sourced from Chengdu Kelong Chemical Reagent Co., Ltd. (Chengdu, China). OE and LE were obtained from Must Biotechnology Co., Ltd. (Chengdu, China). Chromatography-grade methanol and acetonitrile were supplied by Thermo Fisher Scientific Co., Ltd. (Shanghai, China). Furthermore, ultrapure water, essential for the extraction and analysis of OE and LE, was generated using a UPT-T-101 UPT ultrapure water machine fitted with a 0.22 μm filter (Chengdu Ultrapure Water Technology Co., Ltd., Chengdu, China).

### 3.2. Preparation of Deep Eutectic Solvents

[Ch]Cl and the hydrogen bond donors (HBDs), lactic acid, and ethylene glycol, were dried in a vacuum concentrator at 60 °C for 24 h. Subsequently, eight different [Ch]Cl-based DESs were formulated by combining [Ch]Cl as the hydrogen bond acceptor (HBA) with lactic acid and ethylene glycol as the HBDs at molar ratios of 1:1, 1:2, 1:3, and 1:4 (Table 1). The mixtures were subsequently homogenized, utilizing ultrasonic vibration (210 W, 40 °C) until a clear, colorless, and homogeneous liquid was achieved, while different proportions of moisture content were added to DES.

### 3.3. Extraction Procedure

Powdered olive leaves (1 g) were added to a 1000 mL tube. Mechanical wall-breaking extraction (WBE) was conducted, utilizing a multifunctional wall-breaking machine (RBM-769S, Hattiece, Guangzhou, China) in combination with DES. The extraction process was carried out at a stable power of 800 W and a temperature of 25 °C. Subsequently, the impacts of wall-breaking time, water content, and liquid–solid ratio on the OE, OL, and TF were evaluated through single-factor experiments.

### 3.4. Single-Factor Experiments

Preliminary experimental analysis indicated that extraction yields of oleuropein and luteolin-7-O-glucoside did not significantly differ when the temperature was maintained below 60 °C. Consequently, the extraction temperature was set to room temperature (25 °C), and the wall-breaking time was adjusted to a range of 60 to 180 s. Additionally, the water content was varied from 10% to 50%, and the ratio of liquid and solid was adjusted between 100 g/mL and 500 g/mL. Following extraction, the solution was centrifuged at 4000 rpm for 20 min using a H2-16K centrifuge from Shanghai Zhaodi Laboratory Instruments (Shanghai, China). We collected the supernatant for optimization analysis and then processed it through freeze-drying to obtain the EOL. The EOL-rich extracts were sealed and stored at −18 °C for subsequent activity analysis.

### 3.5. Optimization Experiment of Response Surface Methodology

According to the results of the single-factor experiments, the Box–Behnken design (BBD) model with RSM in Design-Expert 20 software was utilized to optimize the extraction conditions for OE, LE and total flavonoids (TF). The BBD, which involves three independent variables (A, wall-breaking time; B, liquid–solid ratio; C, DES moisture content) at three levels (−1, 0, 1), was applied, with the extraction yield (Y) serving as the response value. The coding levels and actual levels of these three factors are presented in Table 8.

### 3.6. HPLC Analysis

OE and LE were detected through high-performance liquid chromatography (HPLC). The analysis was conducted using an Agilent 1260 system (Santa Clara, CA, USA), which was equipped with a ZORBAX Eclipse XDB-C18 (Agilent, Santa Clara, CA, USA) reversed-phase column (5.0 µm, 150 mm × 4.6 mm). The column temperature was maintained at a constant 30 °C. Sample injection was performed with a volume of 10 µL. The mobile phase consisted of eluent A (acetonitrile) and eluent B (a mixture of water and acetic acid in a 99.8/0.2 *v*/*v* ratio), which flowed at a rate of 0.8 mL/min. Detection took place at a wavelength of 240 nm. A gradient elution profile was applied: initially, 16–20% A for the first 5 min, followed by an increase to 20–28% A from 5 to 20 min. Quantification of the extraction yields was carried out using a standard calibration curve, and the results were computed based on the predetermined formula:

$$Extraction\;yields\;(mg/g\;DM)=\frac{Mass\;of\;olive\;leaves\;powder\;\left(g\right)}{Mass\;of\;dried\;crude\;\left(g\right)}\times 100.\;$$

The concentrations of OE and LE are expressed as milligrams of analyte per gram of dry matter (mg/g DM).

### 3.7. Determination of Total Flavonoids

The TFC in the extracts was determined using a modified method from Chlopicka et al. [45]. Briefly, 0.7 mL of supernatant was pipetted into a test tube, followed by the addition of 0.2 mL of 10% aluminum chloride (AlCl_3_) solution and 0.2 mL of 10% sodium nitrite (NaNO_2_) solution. A 2.5 mL aliquot of 1M sodium hydroxide (NaOH) solution was introduced, followed by adjustment of the total volume to 10 mL with distilled water. The absorbance of the resultant solution was then determined at 510 nm using a Thermo Multiskan GO microplate reader (Thermo Fisher Scientific, Waltham, MA, USA). TFC was quantified, with rutin equivalents serving as the standard, using a calibration curve ranging from 0.1 to 1.0 mg/mL. The outcomes were reported in milligrams of rutin equivalent per gram of dry sample weight (mg RE/g).

### 3.8. Determination of Antioxidant Activity

The antioxidant capacities of EOL-rich extracts were evaluated using the DPPH^•^ and ABTS^•+^ methods, due to their extensive utilization in antioxidant assessment [46]. Assay kits, sourced from the Grace Bioengineering Institute located in Suzhou, China, were employed to determine the free radical scavenging activity of the extracts. In the DPPH assay, various concentrations of EOL-rich extracts (40, 60, 80, 100, and 120 mg/mL) were combined with 0.1 mM DPPH^•^ reagent in 1.5 mL tubes and incubated in darkness for 30 min. Subsequently, 80% methanol served as a blank, and vitamin C (Vc) was used as a positive control. Concurrently, pure 70% OE and 50% LE were employed as standard substances, with absorbance measurements taken at 734 nm. For the ABTS^•+^ assay, the same range of EOL-rich extract concentrations was mixed with 0.1 mM ABTS^•+^ reagent and incubated in the dark at room temperature for 6 min. The absorbance was then determined at 734 nm, with anhydrous ethanol serving as the blank and Vc as the positive control. Antioxidant activity for both assays was calculated using the following formula:

$$Antioxidant\;activity\left(\%\right)=1-\;\frac{A-A_{0}}{A_{1}}\times 100\%$$

where *A*, *A*_0_, *A*_1_ denote the absorbance of the sample, blank, and control, respectively.

### 3.9. Determination of Antitumor Activity

The cell lines, namely Eca-109 (esophageal squamous cell carcinoma) and Het-1A (normal esophageal epithelial), were obtained from China West Normal University. The cells were maintained in RPMI-1640 medium, which was supplemented with 10% fetal calf serum and 1% penicillin-streptomycin, and incubated at 37 °C in a 5% CO_2_ atmosphere. Sub-culturing was carried out at a 1:2 dilution ratio upon reaching 80–90% confluence.

For the assessment of cell viability, CCK-8 kits (GBBIO Technologies Inc., Piscataway, NJ, USA) were utilized. A 96-well plate was seeded with cells at a density of 1 × 10^4^ cells per well. The EOL-rich extracts were then prepared by diluting them to concentrations of 25, 50, and 75 μg/mL in RPMI-1640 medium. After a 24-h incubation, 10 µL of CCK-8 solution was introduced into each well, and the plates were incubated for an additional 2 h. Absorbance was measured at 450 nm. Eca-109 and Het-1A cells without treatment served as negative controls. Cell viability was calculated using the formula:

$$Cell\;viability\left(\%\right)=\frac{A-A_{0}}{A_{0}-A_{1}}\times 100\%$$

The absorbance of the well containing cells, samples, and CCK-8 solution is denoted by *A*. *A*_0_ and *A*_1_ represent the absorbance of the wells without cells and without sample solution, respectively.

### 3.10. Statistical Analysis

Optimization of the WBE processes was accomplished using Design-Expert 10 software (employing Response Surface Methodology, RSM, to evaluate interactive effects on response variables). Data processing, figure creation, and analysis were carried out utilizing Origin 20.0 and SPSS Statistics 20.0 software packages. The data underwent statistical evaluation through one-way analysis of variance (ANOVA) to assess variances. Each experiment was performed in triplicate, with three samples per treatment, to ensure reliability. The results are presented as the mean ± standard deviation (SD), with significant differences at the *p* < 0.05 level indicated by different letters.

## 4. Conclusions

Olive leaves are rich in bioactive compounds; we aimed to efficiently extract key active components: OE, LE, and TF. An environmentally friendly DES-based WBE technique was employed, beginning with the screening of eight choline chloride ([Ch]Cl)-based DESs. The optimal DES composition, [Ch]Cl: ethylene glycol (1:3 molar ratio) with 30% moisture, was selected for extraction. Using response surface methodology (RSM), yields of 88.87 mg/g dry matter (DM) for OE, 4.57 mg/g DM for LE, and 114.31 mg RE/g for TF were achieved. The optimal extraction conditions were determined to be 140 s for wall-breaking time, 230 mL/g for liquid-to-solid ratio, and 30% for DES moisture content. Among China’s three primary olive varieties (Arbosana, Arbequina, and Picholine), the young leaves of Picholine contained significantly higher levels of these bioactive components compared to its old leaves. Extracts from Picholine also exhibited notable ABTS^•+^ (155.10 mg/mL) and DPPH^•^ (44.58 mg/mL) radical scavenging activities, although slightly lower than those of pure OE and LE. Furthermore, the extract from all three varieties had a more pronounced effect on cell viability at a concentration of 25 µg/mL after 24 h compared to higher concentrations after 48 h. Notably, Picholine’s extract demonstrated superior antitumor activity by inhibiting the growth of Eca-109 cells and promoting the growth of Het-1A cells at this specific concentration and time frame, outperforming pure OE and LE. In conclusion, our findings indicate that DES-based WBE is an efficient and eco-friendly method for extracting bioactive compounds, particularly OE and flavonoids, from young Picholine olive leaves, which represent a promising source of these valuable compounds.

## Figures and Tables

**Table 1:** Materials and molar ratios of the DES.

No.	Hydrogen Bond Acceptor	Hydrogen Bond Donor	Molar Ratio
DES-1	[Ch]Cl	lactic acid	1:1
DES-2	[Ch]Cl	lactic acid	1:2
DES-3	[Ch]Cl	lactic acid	1:3
DES-4	[Ch]Cl	lactic acid	1:4
DES-5	[Ch]Cl	ethylene glycol	1:1
DES-6	[Ch]Cl	ethylene glycol	1:2
DES-7	[Ch]Cl	ethylene glycol	1:3
DES-8	[Ch]Cl	ethylene glycol	1:4

Note: DES, deep eutectic solvent; [Ch]Cl, choline chloride.

**Table 2:** Changes in OEY, LEY, and TFC from olive leaves using different types of DESs.

No.	OEY (mg/g DM)	LEY (mg/g DM)	TFC (mg RE/g)
DES-1	45.63 ± 0.69 d	3.86 ± 0.06 bc	0.46 ± 0.02 d
DES-2	40.21 ± 0.91 e	3.74 ± 0.02 cd	2.81 ± 0.42 d
DES-3	38.39 ± 0.5 ef	3.84 ± 0.02 bcd	1.42 ± 0.05 d
DES-4	34.89 ± 0.42 f	3.88 ± 0.06 b	1.34 ± 0.10 d
DES-5	53.19 ± 0.73 c	3.73 ± 0.03 d	80.88 ± 0.49 c
DES-6	58.58 ± 0.95 a	4.11 ± 0.05 a	98.82 ± 2.59 a
DES-7	57.92 ± 0.55 ab	3.94 ± 0.05 b	83.05 ± 2.16 c
DES-8	54.28 ± 3.14 bc	3.85 ± 0.04 bcd	90.06 ± 1.11 b

Note: OEY, oleuropein yield; LEY, luteolin-7-O-glucoside yield; TFC, total flavonoid content. Different lowercase letters indicate statistically significant differences on OEY and flavonoids extracted using different DES solvents.

**Table 3:** Independent variables, their levels for the BBD, and the responses obtained.

Run	Extraction Conditions			Extraction Yields		
	WBT (A, s)	LSR (B, mL/g)	DMC (C, %)	OEY (mg/g DM)	LEY (mg/g DM)	TFC (mg RE/g)
1	150 (0)	200 (0)	30 (0)	78.17	3.77	108.61
2	120 (−1)	100 (−1)	30	69.05	2.73	95.10
3	150	200	30	86.71	4.23	114.90
4	150	200	30	74.32	3.46	113.35
5	120	300 (+1)	30	76.82	3.58	93.86
6	150	100	40 (+1)	86.63	4.14	81.92
7	180 (+1)	100	30	81.00	4.22	96.02
8	180	200	40	70.88	3.43	84.33
9	150	300	20 (−1)	76.45	3.58	88.38
10	150	100	20	78.15	3.65	90.23
11	120	200	40	71.94	3.64	85.81
12	150	200	30	73.65	3.51	103.13
13	180	200	20	86.12	4.21	89.46
14	120	200	20	86.27	4.35	94.69
15	150	300	40	86.40	4.27	71.63
16	150	200	30	77.00	3.44	103.05
17	180	300	30	69.49	3.11	67.97

Note: WBT, wall-breaking time; LSR, liquid-solid ratio; DMC, DES moisture content. OEY, oleuropein yield; LEY, luteolin-7-O-glucoside yield; TFC, total flavonoid content. A, wall-breaking time; B, liquid-solid ratio; C, moisture content.

**Table 4:** Polynomial model ANOVA for all independent variables in response surface analysis.

Source	OEY		LEY		TFC	
	F Value	*p*-Value	F Value	*p*-Value	F Value	*p*-Value
Model	463.65	<0.0001 ***	71.38	<0.0001 ***	9.03	0.0042 *
A	105.57	<0.0001 ***	84.97	<0.0001 ***	4.05	0.084
B	66.03	<0.0001 ***	20.34	0.0028 **	6.94	0.0337 *
C	6.03	0.0437 *	23.11	0.0019 **	6.16	0.042 *
AB	8.48	0.0226 *	17.10	0.0044 **	5.81	0.0468 *
AC	256.38	<0.0001 ***	44.33	0.0003 **	0.11	0.746
BC	133.24	<0.0001 ***	5.68	0.0487 *	0.58	0.4729
A^2^	455.53	<0.0001 ***	82.09	<0.0001 ***	7.49	0.0291 *
B^2^	483.92	<0.0001 ***	24.62	0.0016 **	22.82	0.002 **
C^2^	2350.99	<0.0001 ***	305.33	<0.0001 ***	21.65	0.0023 **
Lack of Fit	4.61	0.0871	0.65	0.6252	1.02	0.4736
R^2^	0.9983		0.9892		0.9207	
Adj-R^2^	0.9962		0.9754		0.8188	
CV (%)	0.50		1.92		1.90	

Note: OEY, oleuropein yield; LEY, luteolin-7-O-glucoside yield; TFC, total flavonoid content. R^2^, coefficient of determination; Adj-R^2^, coefficient of determination adjusted; * Significant at *p* < 0.05, ** Significant at *p* < 0.005, *** Significant at *p* < 0.0001.

**Table 5:** Optimal WBE conditions using DES-based extraction.

Factors	OC-OEY	AOC-OEY	OC-LEY	AOC-LEY	OC-TFC	AOC-TFC
Time (s)	141.46	140	141.46	140	143.35	140
Ratio (mL/g)	220.57	230	220.57	230	187.21	230
Moisture (%)	29.68	30	29.68	30	28.09	30
Yield (mg/g)	88.87 ± 1.48		4.57 ± 0.15		114.31 ± 0.22	

Note: OC, optimization conditions; AOC, actual operating conditions. OEY, oleuropein yield; LEY, luteolin-7-O-glucoside yield; TFC, total flavonoid content.

**Table 6:** Analysis of oleuropein and flavonoids in aged and young leaves of major olive cultivars.

Cultivars	Leaf Age	OEY (mg/g DM)	LEY (mg/g DM)	TFC (mg RE/g)
Arbosana	aged leaves	19.50 ± 0.10 c	3.58 ± 0.07 b	52.33 ± 0.98 d
	young leaves	46.48 ± 0.69 b	4.62 ± 0.11 a	55.50 ± 1.02 c
Arbequina	aged leaves	11.79 ± 0.20 e	2.26 ± 0.49 d	27.93 ± 0.62 f
	young leaves	46.59 ± 0.39 b	3.57 ± 0.09 b	63.47 ± 0.93 b
Picholine	aged leaves	14.12 ± 0.13 d	1.72 ± 0.04 e	31.51 ± 1.17 e
	young leaves	52.96 ± 0.89 a	2.91 ± 0.19 c	76.98 ± 1.67 a

Note: OEY, oleuropein yield; LEY, luteolin-7-O-glucoside yield; TFC, total flavonoid content. Results are the mean (±SE) of three independent experiments. Values designated with different letters within columns are statistically different (*p* < 0.05).

**Table 7:** Antioxidant activities of EOL-rich extracts as measured by the ABTS^•^+ and DPPH^•^.

Concentration (mg/mL)	ABTS^•+^					
	Vc	OE	LE	Arbosana	Arbequina	Picholine
20	99.24 ± 2.96 a	99.74 ± 0.13 a	98.39 ± 0.22 a	14.66 ± 0.03 f	16.37 ± 0.69 e	16.51 ± 0.95 f
40	99.43 ± 0.25 a	99.61 ± 0.09 ab	97.87 ± 1.48 a	20.03 ± 1.07 e	17.79 ± 1.57 e	23.11 ± 0.62 e
60	99.62 ± 0.08 a	99.18 ± 0.22 b	98.86 ± 0.51 a	24.01 ± 0.43 d	21.88 ± 0.67 d	24.63 ± 1.69 e
80	98.44 ± 1.84 a	98.55 ± 0.18 c	99.10 ± 0.59 a	26.10 ± 0.38 c	26.48 ± 0.81 c	28.71 ± 0.93 d
100	99.29 ± 0.25 a	98.24 ± 0.12 c	99.05 ± 0.22 a	31.37 ± 1.36 b	31.94 ± 0.85 b	32.70 ± 0.35 c
120	99.19 ± 0.54 a	96.44 ± 0.13 d	98.67 ± 0.79 a	51.78 ± 1.81 a	56.76 ± 2.32 a	58.14 ± 2.22 b
EC50	3.59	1.15	2.41	180.97	163.68	155.10
	**DPPH^•^**					
20	95.70 ± 0.77 a	82.52 ± 1.84 d	89.59 ± 2.72 c	26.38 ± 0.85 f	36.88 ± 1.11 e	31.31 ± 1.16 f
40	95.45 ± 0.46 a	87.35 ± 3.97 c	93.99 ± 0.09 a	32.59 ± 1.30 e	45.78 ± 3.03 d	48.16 ± 1.64 e
60	95.88 ± 0.46 a	93.06 ± 0.56 b	92.48 ± 0.53 ab	47.17 ± 1.36 d	83.13 ± 1.85 ab	48.11 ± 2.31 e
80	95.20 ± 0.18 a	99.09 ± 0.41 a	92.13 ± 1.28 abc	52.21 ± 1.95 c	63.91 ± 2.38 c	64.03 ± 2.15 c
100	95.20 ± 0.49 a	97.12 ± 1.91 a	90.69 ± 1.10 bc	56.27 ± 0.74 b	80.56 ± 0.37 b	75.11 ± 1.63 b
120	94.71 ± 1.38 a	96.16 ± 1.09 ab	94.51 ± 1.69 a	68.14 ± 0.77 a	86.47 ± 1.67 a	82.47 ± 1.95 a
EC50	2.76	5.97	1.37	68.82	33.43	44.58

Note:Vc, vitamin C; OE, oleuropein; LE, luteolin-7-O-glucoside. Statistically significant differences exist in Antioxidant activities of the same component at different concentration gradients, which are indicated by different lowercase letters.

**Table 8:** Independent variables and their levels for Box–Behnken design.

Independent Variable	Symbols	Factor Level		
		−1	0	1
Wall-breaking time (s)	A	120	150	180
Liquid–solid ratio (mL/g)	B	100	200	300
DES moisture content (%)	C	20	30	40

## Data Availability

The data that support the findings of this study are available from the corresponding author upon reasonable request.
